# Lymphocyte–C-reactive protein ratio can differentiate disease severity of COVID-19 patients and serve as an assistant screening tool for hospital and ICU admission

**DOI:** 10.3389/fimmu.2022.957407

**Published:** 2022-09-23

**Authors:** Jian-Nan Zhang, Yang Gao, Xin-Tong Wang, Na-Na Li, Xue Du, Yu-Jia Tang, Qi-Qi Lai, Peng-Fei Chen, Chuang-Shi Yue, Ji-Han Wu, Kai Kang, Ming-Yan Zhao

**Affiliations:** ^1^ Department of Critical Care Medicine, The First Affiliated Hospital of Harbin Medical University, Harbin, China; ^2^ Department of Critical Care Medicine, The Sixth Affiliated Hospital of Harbin Medical University, Harbin, China; ^3^ Institute of Critical Care Medicine, The Sino Russian Medical Research Center of Harbin Medical University, Harbin, China

**Keywords:** lymphocyte–C-reactive protein ratio, disease severity, COVID-19, SARS-CoV-2 infection, screening tool, hospital admission, ICU admission

## Abstract

In this study, we aimed to explore whether lymphocyte–C-reactive protein ratio (LCR) can differentiate disease severity of coronavirus disease 2019 (COVID-19) patients and its value as an assistant screening tool for admission to hospital and intensive care unit (ICU). A total of 184 adult COVID-19 patients from the COVID-19 Treatment Center in Heilongjiang Province at the First Affiliated Hospital of Harbin Medical University between January 2020 and March 2021 were included in this study. Patients were divided into asymptomatic infection group, mild group, moderate group, severe group, and critical group according to the Diagnosis and Treatment of New Coronavirus Pneumonia (ninth edition). Demographic and clinical data including gender, age, comorbidities, severity of COVID-19, white blood cell count (WBC), neutrophil proportion (NEUT%), lymphocyte count (LYMPH), lymphocyte percentage (LYM%), red blood cell distribution width (RDW), platelet (PLT), C-reactive protein (CRP), alanine aminotransferase (ALT), aspartate aminotransferase (AST), serum creatinine (SCr), albumin (ALB), total bilirubin (TB), direct bilirubin (DBIL), indirect bilirubin (IBIL), and D-dimer were obtained and collated from medical records at admission, from which sequential organ failure assessment (SOFA) score and LCR were calculated, and all the above indicators were compared among the groups. Multiple clinical parameters, including LYMPH, CRP, and LCR, showed significant differences among the groups. The related factors to classify COVID-19 patients into moderate, severe, and critical groups included age, number of comorbidities, WBC, LCR, and AST. Among these factors, the number of comorbidities showed the greatest effect, and only WBC and LCR were protective factors. The area under the receiver operating characteristic (ROC) curve of LCR to classify COVID-19 patients into moderate, severe, and critical groups was 0.176. The cutoff value of LCR and the sensitivity and specificity of the ROC curve were 1,780.7050 and 84.6% and 66.2%, respectively. The related factors to classify COVID-19 patients into severe and critical groups included the number of comorbidities, PLT, LCR, and SOFA score. Among these factors, SOFA score showed the greatest effect, and LCR was the only protective factor. The area under the ROC curve of LCR to classify COVID-19 patients into severe and critical groups was 0.106. The cutoff value of LCR and the sensitivity and specificity of the ROC curve were 571.2200 and 81.3% and 90.0%, respectively. In summary, LCR can differentiate disease severity of COVID-19 patients and serve as a simple and objective assistant screening tool for hospital and ICU admission.

## Background

Although it has been more than 2 years since the first outbreak of coronavirus disease 2019 (COVID-19) in Wuhan, this highly pathogenic disease has so far shown no signs of abating, suggesting that severe acute respiratory syndrome coronavirus type 2 (SARS-CoV-2) may coexist with humans for a long time to come (1). As the epidemic spreads, rapid recombination and successive mutation of SARS-CoV-2 make its variants more pathogenic and transmissible, which in turn possess the ability to continue spreading in the face of rising population immunity (2–5). Without predictable preparation and effective medical management, the rapid outbreak and surge of COVID-19 patients in a short period will overwhelm limited medical resources (6). Under these new circumstances, China’s response must strike a balance between epidemic prevention and management and economic development, making the need for China to adopt a unique anti-epidemic path in the near future, rather than copying the models in other countries.

The latest Diagnosis and Treatment of New Coronavirus Pneumonia (ninth edition) has pointed out that mild COVID-19 cases, let alone asymptomatic infection cases, only need centralized isolation without hospitalization. The timely detection of COVID-19 patients with disease progression to severe and critical illness and their transfer to intensive care unit (ICU) for appropriate organ support are expected to eventually improve prognosis (7). There is an urgent need to explore an easy-to-implement and high-accuracy clinical objective parameter as an assistant screening tool to identify COVID-19 patients who need hospitalization and detect severe and critical COVID-19 patients who need timely transfer to ICU. The pathogenesis of COVID-19 may be complex and related to inflammation, immunity, blood coagulation, multiple organ functions, and their interactions (8–10). Therefore, the ideal candidate clinical variables should contain some parameters reflecting these aspects but should not be too complicated and difficult to implement as an assistant screening tool for hospital and ICU admission.

To address the above issue, we aimed to explore whether the lymphocyte–C-reactive protein ratio (LCR) can differentiate disease severity of COVID-19 patients and its value as an assistant screening tool for hospital and ICU admission. Our findings will provide a solid basis for the rational allocation and utilization of limited medical resources in the ongoing pandemic.

## Methods

### Study design

A total of 184 adult COVID-19 patients from the COVID-19 Treatment Center in Heilongjiang Province at the First Affiliated Hospital of Harbin Medical University between January 2020 and March 2021 were included in this single-center retrospective study. These patients were divided into asymptomatic infection group (*n* = 16), mild group (*n* = 23), moderate group (*n* = 105), severe group (*n* = 33), and critical group (*n* = 7) according to the Diagnosis and Treatment of New Coronavirus Pneumonia (ninth edition). Demographic and clinical data including gender, age, comorbidities, severity of COVID-19, white blood cell count (WBC), neutrophil proportion (NEUT%), lymphocyte count (LYMPH), lymphocyte percentage (LYM%), red blood cell distribution width (RDW), platelet (PLT), C-reactive protein (CRP), alanine aminotransferase (ALT), aspartate aminotransferase (AST), serum creatinine (SCr), albumin (ALB), total bilirubin (TB), direct bilirubin (DBIL), indirect bilirubin (IBIL), and D-dimer were obtained and collated from medical records at admission. From these data, sequential organ failure assessment (SOFA) score and LCR were calculated, and all the above indicators were compared among the groups. This study was reviewed and approved by the Ethics Committee of the First Affiliated Hospital of Harbin Medical University (IRB number: IRB-AF/SC-04/01.0).

### Study population

**Figure 1:**
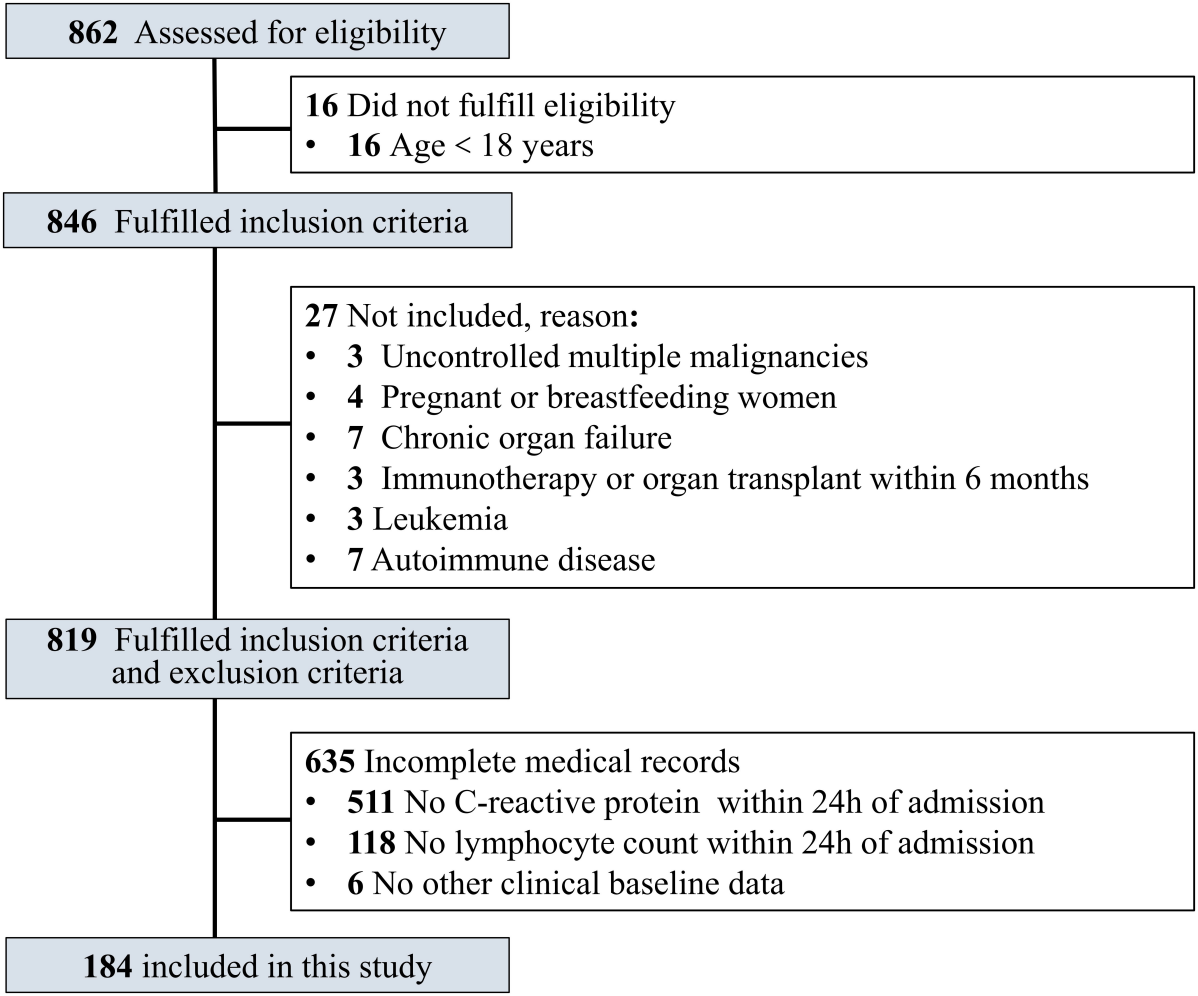
Flowchart of the study participants.

In this study, the inclusion criteria were age ≥18 years old and confirmed COVID-19 patients. COVID-19 patients who met the following criteria were excluded: uncontrolled malignant tumors with multiple metastases, leukemia, acquired immunodeficiency syndrome (AIDS), chronic organ failure, immunotherapy or organ transplant within 6 months, autoimmune disorder, pregnant or breastfeeding women, and incomplete medical records (**Figure 1**).

### Diagnosis and classification of COVID-19

All enrolled adult COVID-19 patients were confirmed by detection of SARS-CoV-2 nucleic acids on oropharyngeal swabs, nasopharyngeal swabs, or lower respiratory tract specimens. These patients were then classified into asymptomatic infection group, mild group, moderate group, severe group, and critical group according to the Diagnosis and Treatment of New Coronavirus Pneumonia (ninth edition).

### Data collection

Demographic data, involving gender, age, comorbidities, and clinical data, including the severity of COVID-19, SOFA score, WBC, NEUT%, LYMPH, LYM%, RDW, PLT, CRP, LCR, ALT, AST, SCr, ALB, TB, DBIL, IBIL, and D-dimer were obtained and collated from medical records at admission by dedicated personnel in our research team. None of the other members of our research team was privy to the enrolled patient’s personal information beyond what was required for this study.

### Statistical analyses

SPSS 23.0 (SPSS, Inc., Chicago, IL, USA) was used for statistical analyses. Continuous data with normal distribution are expressed as mean ± standard deviation (SD), while those with non-normal distribution are described as median (P25, P75). One-way analysis of variance (ANOVA) was adopted for three-group comparisons of continuous data with normal distribution. If there was a significant difference, the least significant difference (LSD) method was used for further pairwise comparison. The Kruskal–Wallis rank-sum test was employed for three-group comparisons and further pairwise comparisons of continuous data with non-normal distribution. Independent-samples *t*-test was used to perform intergroup comparisons of continuous data with normal distribution, while the Mann–Whitney *U* test was adopted for intergroup comparisons of continuous data with non-normal distribution. On the basis of intergroup comparisons, multiple logistic regression analysis was conducted with significant variables as independent variables and groups as dependent variables. The independent variables were screened by the Wald backward method. The receiver operating characteristic (ROC) curve of LCR was analyzed, and the area under the ROC curve, the sensitivity and specificity of the ROC curve, and the cutoff value of LCR were calculated. *P*-values <0.05 were considered to indicate statistical significance.

## Results

### Comparison of demographic and clinical baseline data among asymptomatic infection and mild groups combined, moderate group, and severe and critical groups combined

A total of 184 adult COVID-19 patients from the COVID-19 Treatment Center in Heilongjiang Province at the First Affiliated Hospital of Harbin Medical University between January 2020 and March 2021 were included and divided into asymptomatic infection group (*n* = 16), mild group (*n* = 23), moderate group (*n* = 105), severe group (*n* = 33), and critical group (*n* = 7) according to the Diagnosis and Treatment of New Coronavirus Pneumonia (ninth edition). Age, number of comorbidities, WBC, NEUT%, LYMPH, LYM%, PLT, CRP, AST, ALB, TB, IBIL, D-dimer, LCR, and SOFA score showed significant differences among the groups (*P* = 0.000, *P* = 0.000, *P* = 0.021, *P* = 0.000, *P* = 0.000, *P* = 0.000, *P* = 0.000, *P* = 0.000, *P* = 0.000, *P* = 0.000, *P* = 0.000, *P* = 0.014, *P* = 0.000, *P* = 0.000, *P* = 0.000, respectively). No significant differences were observed in the remaining demographic and clinical baseline data, including gender, hypertension, diabetes, RDW, ALT, SCr, and DBIL (**Table 1**).

**Table 1:** Comparison of demographic and clinical baseline data among asymptomatic infection and mild groups combined, moderate group, and severe and critical groups combined.

	Asymptomatic infection and mild groups combined	Moderate group	Severe and critical groups combined	*Z*/*F*/*χ* ^2^	*P*
Age	42.00 (31.00, 56.00)	62.00 (48.00, 67.00) a	68.00 (58.25, 75.75) ab	37.382	0.000
Gender (F/M)	19/20	61/44	22/18	1.016	0.602
Hypertension (Y/N)	6/33	38/67	14/26	5.989	0.052
Diabetes (Y/N)	1/38	15/90	8/32	5.626	0.060
Number of comorbidities	0 (0, 1)	1 (1, 3) a	3 (1, 4.75) ab	36.277	0.000
WBC	6.38 ± 2.04	5.29 ± 2.03 a	5.36 ± 2.47 a	3.930	0.021
NEUT%	62.37 ± 10.40	61.40 ± 11.92	73.63 ± 17.25 ab	13.397	0.000
LYMPH	1.50 (1.19, 1.83)	1.24 (0.89, 1.63)	0.65 (0.42, 0.99) ab	45.062	0.000
LYM%	25.37 ± 9.27	27.37 ± 9.85	15.85 ± 8.99 ab	21.281	0.000
RDW	40.20 (38.70, 42.20)	41.00 (38.40, 43.15)	41.55 (38.00, 43.93)	0.282	0.868
PLT	228.00 (177.00, 271.00)	174.00 (140.00, 228.00) a	148.00 (121.50, 209.25) a	16.799	0.000
CRP	4.57 ± 8.03	21.23 ± 38.73 a	52.76 ± 47.98 ab	17.474	0.000
ALT	23.60 (17.70, 36.89)	25.60 (17.50, 36.75)	28.61 (18.15, 38.70)	0.660	0.791
AST	19.50 (16.80, 24.83)	25.60 (19.36, 37.65) a	32.94 (20.50, 51.67) a	20.579	0.000
SCr	61.20 (48.99, 73.88)	62.25 (53.20, 73.75)	63.79 (51.10, 86.28)	0.376	0.829
ALB	42.93 ± 4.63	39.74 ± 5.00 a	34.85 ± 5.75 ab	25.551	0.000
TB	8.80 (4.55, 10.80)	7.80 (6.13, 11.71)	11.00 (7.74, 16.41) ab	15.929	0.000
DBIL	1.70 (0.00, 2.30)	2.00 (0.00, 3.10)	2.65 (0.00, 4.68)	5.685	0.058
IBIL	7.00 (3.13, 8.80)	6.74 (4.30, 8.81)	8.46 (5.67, 12.48) ab	8.519	0.014
D-dimer	0.56 (0.47, 0.72)	0.74 (0.54, 1.01) a	1.17 (0.67, 3.01) ab	27.192	0.000
LCR	9,554.14 (2,560.61, 27,000.00)	1,404.96 (575.52, 4,941.62) a	140.42 (71.32, 420.27) ab	76.498	0.000
SOFA score	0 (0, 0)	0 (0, 1)	3 (2, 5) ab	80.893	0.000

a and b represent significant differences compared to asymptomatic infection and mild groups combined and moderate group, respectively.

### Comparison of demographic and clinical baseline data between asymptomatic infection and mild groups combined and moderate, severe, and critical groups combined

Age, hypertension, diabetes, number of comorbidities, WBC, LYMPH, PLT, CRP, AST, ALB, DBIL, D-dimer, LCR, and SOFA score showed significant differences between the groups (*P* = 0.000, *P* = 0.015, *P* = 0.029, *P* = 0.000, *P* = 0.001, *P* = 0.000, *P* = 0.000, *P* = 0.000, *P* = 0.000, *P* = 0.000, *P* = 0.033, *P* = 0.000, *P* = 0.000, *P* = 0.000, respectively), while no significant differences were seen in the remaining demographic and clinical baseline data, including gender, NEUT%, LYM%, RDW, ALT, SCr, TB, and IBIL (**Table 2**).

**Table 2:** Comparison of demographic and clinical baseline data between asymptomatic infection and mild groups combined and moderate, severe, and critical groups combined.

	Asymptomatic infection and mild groups combined	Moderate, severe, and critical groups combined	*Z*/*t*/*χ* ^2^	*P*
Age	42.00 (31.00, 56.00)	64.00 (50.50, 70.00)	−5.335	0.000
Gender (F/M)	19/20	83/62	0.904	0.342
Hypertension (Y/N)	6/33	52/93	5.970	0.015
Diabetes (Y/N)	1/38	23/122	4.792	0.029
Number of comorbidities	0 (0, 1)	2 (1, 3)	−5.058	0.000
WBC	5.98 (4.92, 7.46)	5.00 (3.85, 6.26)	−3.302	0.001
NEUT%	62.37 ± 10.40	64.77 ± 14.61	−0.963	0.337
LYMPH	1.50 (1.19, 1.83)	1.07 (0.67, 1.59)	−3.688	0.000
LYM%	25.37 ± 9.27	24.19 ± 10.89	0.619	0.537
RDW	40.20 (38.70, 42.20)	41.00 (38.35, 43.15)	−0.422	0.673
PLT	228.00 (177.00, 271.00)	168.00 (138.00, 224.00)	−3.722	0.000
CRP	1.68 (0.50, 6.02)	13.98 (3.94, 34.87)	−6.137	0.000
ALT	23.60 (17.70, 36.89)	27.00 (17.63, 36.90)	−0.608	0.543
AST	19.50 (16.80, 24.83)	27.30 (19.80, 42.04)	−4.162	0.000
SCr	62.20 (48.99, 73.88)	62.30 (52.35, 74.20)	−0.097	0.923
ALB	42.93 ± 4.63	38.39 ± 5.64	4.623	0.000
TB	8.80 (4.55, 10.80)	8.80 (6.55, 13.35)	−1.690	0.091
DBIL	1.70 (0.00, 2.30)	2.10 (0.00, 3.35)	−2.138	0.033
IBIL	7.00 (3.13, 8.80)	7.09 (4.40, 9.69)	−1.128	0.259
D-dimer	0.56 (0.47, 0.72)	0.80 (0.56, 1.24)	−4.031	0.000
LCR	9,554.14 (2,560.61, 27,000.00)	733.77 (241.72, 3,629.01)	−6.211	0.000
SOFA score	0 (0, 0)	1 (0, 2)	−4.500	0.000

### Analysis of the related factors to classify COVID-19 patients into moderate, severe, and critical groups

**Figure 2:**
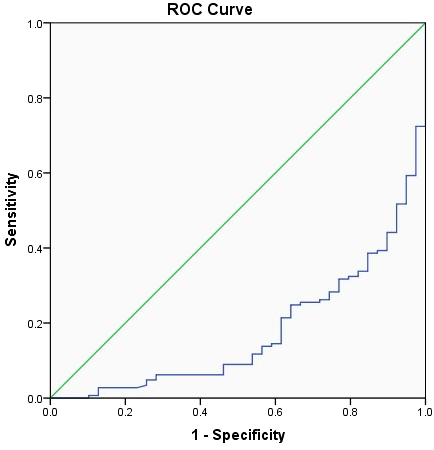
The receiver operating characteristic (ROC) curve of LCR to classify COVID-19 patients into moderate, severe, and critical groups.

The related factors to classify COVID-19 patients into moderate, severe, and critical groups included age, number of comorbidities, WBC, LCR, and AST. With every unit change in age, number of comorbidities, WBC, LCR, and AST, the possibility of classifying COVID-19 patients into moderate, severe, and critical groups was 1.054, 1.814, 0.719, 0.9999, and 1.064 of the original rates, respectively. Among these factors, the number of comorbidities showed the greatest effect, and only WBC and LCR were protective factors (**Table 3**). The area under the ROC curve of LCR to classify COVID-19 patients into moderate, severe, and critical groups was 0.176 (**Figure 2** and **Table 4**). The cutoff value of LCR and the sensitivity and specificity of the ROC curve were 1,780.7050 and 84.6% and 66.2%, respectively.

**Table 3:** Analysis of the related factors to classify COVID-19 patients into moderate, severe, and critical groups.

	*B*	SE	Wald	*P*-value	OR	95% CI	
						Lower bound	Upper bound
Age	0.052	0.017	9.088	0.003	1.054	1.019	1.090
Number of comorbidities	0.595	0.265	5.038	0.025	1.814	1.078	3.051
WBC	−0.331	0.129	6.609	0.010	0.719	0.558	0.924
LCR	−0.00089	0.00025	12.359	0.000	0.9999	0.99986	0.99996
AST	0.062	0.027	5.335	0.021	1.064	1.009	1.122
Constant	−1.081	1.169	0.855	0.355	0.339		

**Table 4:** Area under the receiver operating characteristic curve.

Area	SE	Asymptotic, *P*-value	Lower bound	Asymptotic 95% CI	
				Upper bound
0.176	0.034	0.000	0.108	0.243

### Comparison of demographic and clinical baseline data between asymptomatic infection, mild, and moderate groups combined and severe and critical groups combined

Significant differences were obtained in age, number of comorbidities, NEUT%, LYMPH, LYM%, PLT, CRP, AST, ALB, TB, IBIL, D-dimer, LCR, and SOFA score between the groups (*P* = 0.000, *P* = 0.000, *P* = 0.000, *P* = 0.000, *P* = 0.000, *P* = 0.008, *P* = 0.000, *P* = 0.004, *P* = 0.000, *P* = 0.000, *P* = 0.004, *P* = 0.000, *P* = 0.000, *P* = 0.000, respectively), while no significant differences were observed in the remaining demographic and clinical baseline data, including gender, hypertension, diabetes, WBC, RDW, ALT, SCr, and DBIL (**Table 5**).

**Table 5:** Comparison of demographic and clinical baseline data between asymptomatic infection, mild, and moderate groups combined and severe and critical groups combined.

	Asymptomatic infection, mild, and moderate groups combined	Severe and critical groups combined	*Z*/*t*/*χ* ^2^	*P*
Age	55.50 (42.25, 66.00)	68.00 (58.25, 75.75)	−4.331	0.000
Gender (F/M)	80/64	22/18	0.004	0.950
Hypertension (Y/N)	44/100	14/26	0.286	0.592
Diabetes (Y/N)	16/128	8/32	2.181	0.140
Number of comorbidities	1 (0, 2)	3 (1, 4.75)	−4.529	0.000
WBC	5.27 (3.99, 6.78)	4.85 (3.79, 5.87)	−1.225	0.221
NEUT%	61.55 (53.95, 69.33)	75.8 (66.95, 85.08)	−5.249	0.000
LYMPH	1.35 (0.99, 1.69)	0.65 (0.42, 0.99)	−6.403	0.000
LYM%	26.82 ± 9.71	15.85 ± 8.99	6.424	0.000
RDW	40.8 (38.50, 42.88)	41.55 (38.00, 43.93)	−0.426	0.670
PLT	198.00 (149.75, 238.75)	148.00 (121.50, 209.25)	−2.668	0.008
CRP	6.30 (1.70, 14.48)	35.49 (20.90, 68.05)	−7.012	0.000
ALT	25.37 (17.55, 36.78)	28.61 (18.15, 38.70)	−0.685	0.494
AST	23.36 (18.00, 34.17)	32.94 (20.50, 51.67)	−2.873	0.004
SCr	62.08 (52.45, 73.16)	63.79 (51.10, 86.28)	−0.609	0.542
ALB	40.60 ± 5.09	34.85 ± 5.75	6.152	0.000
TB	8.10 (5.93, 10.98)	11.00 (7.74, 16.41)	−3.940	0.000
DBIL	1.85 (0.00, 3.00)	2.65 (0.00, 4.68)	−1.600	0.110
IBIL	6.77 (4.20, 8.78)	8.455 (5.67, 12.48)	−2.898	0.004
D-dimer	0.69 (0.52, 0.94)	1.17 (0.67, 3.01)	−4.284	0.000
LCR	2,407.47 (640.31, 8,755.06)	140.42 (71.32, 420.27)	−7.621	0.000
SOFA score	0 (0, 1)	3 (2, 5)	−8.721	0.000

### Analysis of the related factors to classify COVID-19 patients into severe and critical groups

**Figure 3:**
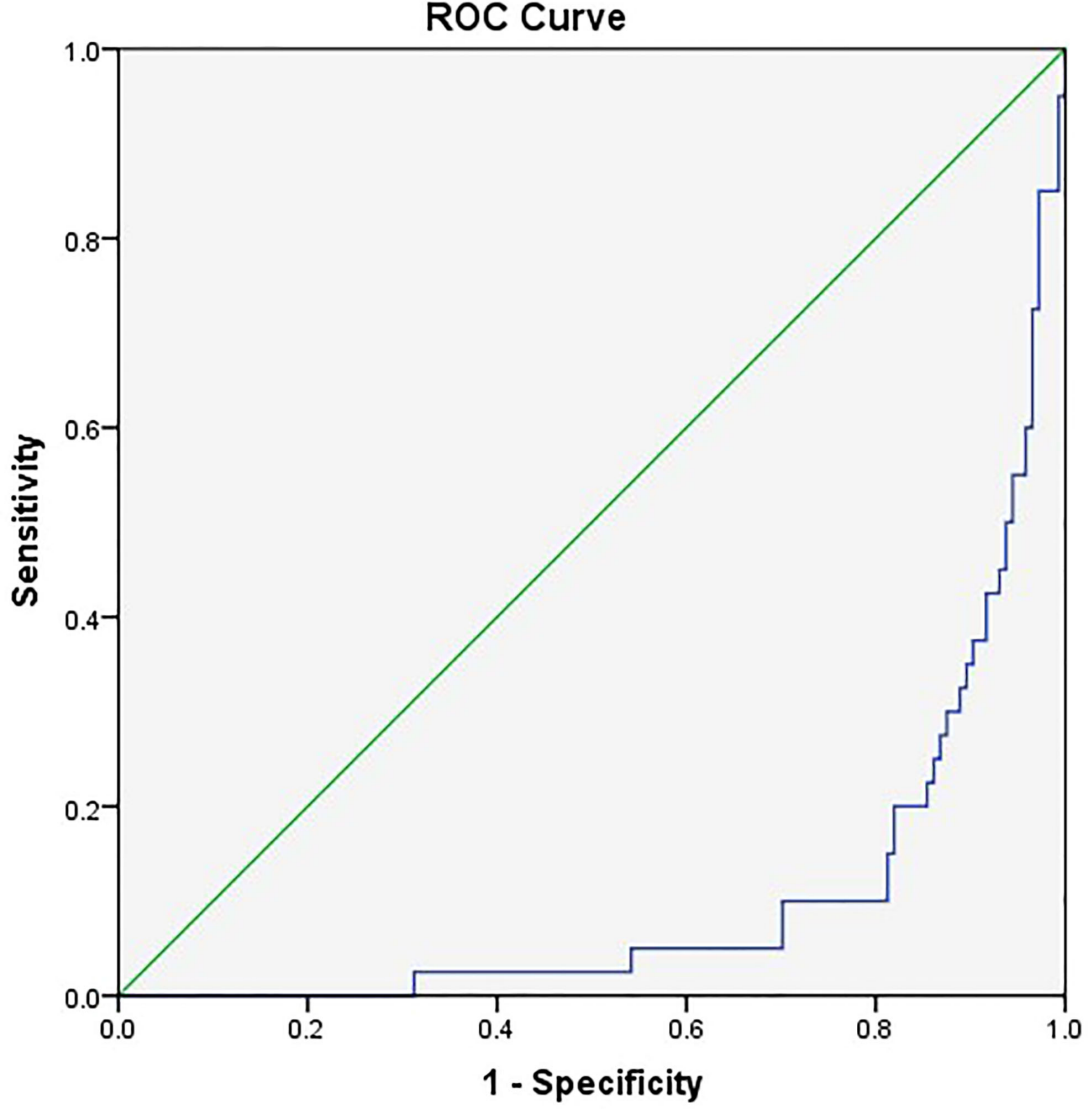
The receiver operating characteristic (ROC) curve of LCR to classify COVID-19 patients into severe and critical groups.

The related factors to classify COVID-19 patients into severe and critical groups included the number of comorbidities, PLT, LCR, and SOFA score. With every unit change in the number of comorbidities, PLT, LCR, and SOFA score, the possibility of classifying COVID-19 patients into severe and critical groups was 1.466, 1.009, 0.9996, and 3.56 of the original rates, respectively. Among these factors, SOFA score showed the greatest effect, and only LCR was a protective factor (**Table 6**). The area under the ROC curve of LCR to classify COVID-19 patients into severe and critical groups was 0.106 (**Figure 3** and **Table 7**). The cutoff value of LCR and the sensitivity and specificity of the ROC curve were 571.2200 and 81.3% and 90.0%, respectively.

**Table 6:** Analysis of the related factors to classify COVID-19 patients into severe and critical groups.

	*B*	SE	Wald	*df*	*P*-value	OR	95% CI	
							Lower bound	Upper bound
Number of comorbidities	0.382	0.180	4.527	1.000	0.033	1.466	1.031	2.084
PLT	0.009	0.004	4.002	1.000	0.045	1.009	1.000	1.018
LCR	−0.00039	0.00020	3.847	1.000	0.049	0.99960	0.99920	0.99999
SOFA score	1.270	0.256	24.542	1.000	0.000	3.560	2.154	5.884
Constant	−4.881	1.107	19.438	1.000	0.000	0.008		

**Table 7:** Area under the receiver operating characteristic curve.

Area	SE	Asymptotic, *P*-value	Asymptotic 95% CI	
			Lower bound	Upper bound
0.106	0.026	0.000	0.054	0.157

## Discussion

SARS-CoV-2, a novel highly pathogenic human coronavirus (hCoV), has posed an unprecedented and persistent threat to the global health system due to rapid evolution resulting in constant emergence of variants with spike protein mutations. Mutations in SARS-CoV-2 enable it to facilitate antigenic shifting for escaping the host antiviral defense and the humoral immune system. This makes it possible to break through the protection of vaccination and then increases the risk of infection in vaccinated populations and reinfection in those previously infected with different variants and even leads to repeated outbreaks of the epidemic (3, 11–13). Although human-to-human transmission is still the main pathway of COVID-19 spread, SARS-CoV-2 infections caused by indirect transmission from the contamination of inert/inanimate surfaces are increasingly common (14). It is important to note that environmental contamination caused by infected animals, especially domestic pets, constitutes a potential virus reservoir and may be one of the pathways of SARS-CoV-2 transmission during COVID-19 outbreaks, despite the lack of direct evidence of human–animal–human transmission (15, 16). A small blind spot in epidemic prevention and management will lead to a total loss of the epidemic response (17).

COVID-19 patients with different disease severity should be triaged to ensure the rational allocation and utilization of limited medical resources, such as centralized isolation and treatment for asymptomatic infection and mild patients, hospitalization for moderate patients, and timely transfer to ICU for severe and critical cases. Therefore, an easy-to-implement and high-accuracy clinical objective parameter as an assistant screening tool to classify COVID-19 patients is required. Multiple candidate clinical parameters, including LYMPH and CRP involved in this study, have been shown to be closely associated with disease severity, rapid progression, and clinical prognosis in COVID-19 patients (10, 18–21).

Lymphocytes are a type of white blood cell, accounting for about 20%–40% of all white blood cells. They are mainly produced by lymphoid organs and reflect the body’s protective immune ability against infection. Related studies showed that lymphopenia at the time of admission was strongly associated with organ damage, disease severity, need for intensive therapy, and poor prognosis in COVID-19 patients, especially in younger cases (22, 23). Redistribution of T cells, termination of T-cell activation by proinflammatory cytokines, and direct destruction of T cells induced by SARS-CoV-2 might be the main causes of lymphopenia (21, 24–26). Likewise, CRP, as a non-specific acute-phase reactant and representative marker of systemic inflammatory response syndrome (SIRS), was found to be significantly elevated at the initial and progression stages of SARS-CoV-2 infection and could be an early clinical predictor for disease severity and adverse outcomes (18, 27, 28). CRP could also activate complement and co-induce the production and release of proinflammatory cytokines, which were involved in the pathogenesis of COVID-19 (29). This further suggested that uncontrolled systemic inflammatory response to SARS-CoV-2 invasion, rather than the fatal virus infection itself, is a driver behind disease deterioration and poor prognosis in COVID-19 patients (8).

It is self-evident that imbalanced immune responses and uncontrolled systemic inflammatory responses intertwine, interacting and influencing each other in the pathogenesis of COVID-19 (30). LCR, as the ratio of the above two clinical parameters, represents both the protective immune activation status and the degree of systemic inflammatory response of the body after SARS-CoV-2 invasion. Perioperative LCR was first utilized as a promising novel biomarker for assessing disease development and the risk of recurrence. It was used in predicting postoperative complications and short-term and long-term prognoses and determining individualized therapeutic strategies in patients with malignant diseases and then extended to many other areas (31–34). A declined level of LCR in COVID-19 patients suggests compromised immune responses and/or an enhancement of systemic inflammatory responses, either of which can be fatal after SARS-CoV-2 invasion. Several lines of evidence demonstrated that the level of LCR could provide pivotal information for evaluating disease severity and predicting prognosis in COVID-19 patients, but its value proposed in our study as an assistant screening tool for admission to hospital and ICU had not been further explored (35, 36).

To our knowledge, our study was the first to explore the value of LCR as an assistant screening tool for COVID-19-related hospital and ICU admission. Our results showed that LCR exhibited significant differences among the groups regardless of grouping. LCR was a protective factor in moderate, severe, and critical groups combined, as well as severe and critical groups combined. The cutoff values of LCR to classify COVID-19 patients into moderate, severe, and critical groups and severe and critical groups were 1,780.7050 and 571.2200, respectively, proving that it can be utilized as an assistant screening tool to triage COVID-19 patients. That is, when the level of LCR in COVID-19 patients is greater than 1,780.7050, between 178.7050 and 571.2200, and less than 571.2200, centralized isolation and treatment, hospitalization, and timely transfer to ICU can be considered, respectively.

Several limitations in the present study should be noted. First of all, the reliability and generalizability of our conclusion were limited by the nature of a single-center retrospective study. Second, due to the lack of LYMPH and CRP within 24 h of admission in a large number of asymptomatic infection and mild patients with COVID-19, the final sample size of our study was relatively small, and thus the results need to be interpreted with caution. Finally, the clinical value of dynamic monitoring of LCR was not further discussed.

## Conclusion

The present study is the first pilot study showing that LCR, as the ratio of lymphocyte to CRP, can differentiate disease severity of COVID-19 patients and serve as a simple and objective assistant screening tool for admission to hospital and ICU. Our findings need to be further validated with a larger sample size.

## Data availability statement

The raw data supporting the conclusions of this article will be made available by the authors, without undue reservation.

## Ethics statement

The study was reviewed and approved by the Ethics Committee of the First Affiliated Hospital of Harbin Medical University (IRB-AF/SC-04/01.0). Written informed consent for participation was not required for this study in accordance with national legislation and institutional requirements.

## Author contributions

J-NZ, YG, X-TW, N-NL, XD, KK, and M-YZ took part in the literature search, conception, study design, statistical analysis, analysis and discussion of results, and manuscript preparation, editing, and review. Y-JT, Q-QL, P-FC, C-SY, and J-HW assisted in the literature search, data acquisition and collation, statistical analysis, analysis and discussion of results, and manuscript preparation. All authors read and approved the final article. J-NZ, YG, X-TW, N-NL and XD contributed equally to this work.

## Funding

This study was supported by the Key Research and Development Project of Heilongjiang Province (GA21C011), the National Natural Science Foundation of China (Nos. 81772045, 81902000, and 82172164), Heilongjiang Province Postdoctoral Start-up Fund (LBH-Q20037), and Scientific Research Innovation Fund of The First Affiliated Hospital of Harbin Medical University (Nos. 2021M08).

## Acknowledgments

The authors are grateful to all colleagues who worked with them in the COVID-19 Treatment Center of Heilongjiang Province and all those who provided selfless advice and help for this article. We pay tribute to the medical staff who lost their lives in the national fight against the COVID-19 epidemic.

## Conflict of interest

The authors declare that the research was conducted in the absence of any commercial or financial relationships that could be construed as a potential conflict of interest.

## Publisher’s note

All claims expressed in this article are solely those of the authors and do not necessarily represent those of their affiliated organizations, or those of the publisher, the editors and the reviewers. Any product that may be evaluated in this article, or claim that may be made by its manufacturer, is not guaranteed or endorsed by the publisher.
